# Antidiabetic, Antimicrobial, and Molecular Profiling of Selected Medicinal Plants

**DOI:** 10.1155/2021/5510099

**Published:** 2021-05-06

**Authors:** Babita Aryal, Purushottam Niraula, Karan Khadayat, Bikash Adhikari, Dadhiram Khatri Chhetri, Basanta Kumar Sapkota, Bibek Raj Bhattarai, Niraj Aryal, Niranjan Parajuli

**Affiliations:** ^1^Biological Chemistry Lab, Central Department of Chemistry, Tribhuvan University, Kirtipur, Nepal; ^2^Pharmaceutical Institute, Department of Pharmaceutical Biology, University of Tübingen, Tübingen, Germany

## Abstract

Natural products have been the center of attraction ever since they were discovered. Among them, plant-based natural products were popular as analgesics, anti-inflammatory, antidiabetic, and cosmetics and possess widespread biotechnological applications. The use of plant products as cosmetics and therapeutics is deep-rooted in Nepalese society. Although there are few ethnobotanical studies conducted, extensive research of these valuable medicinal plants has not been a priority due to the limitation of technology and infrastructure. Here, we selected 4 traditionally used medicinal plants to examine their bioactive properties and their enzyme inhibition potential. *α*-Glucosidase and *α*-amylase inhibitory activities were investigated using an *in vitro* model followed up by antioxidant and antimicrobial activities. The present study shows that ethyl acetate fraction of *Melastoma melabathrium* (IC_50_ 9.1 ± 0.3 *µ*g/mL) and water fraction *Acacia catechu* (IC_50_ 9.0 ± 0.6 *µ*g/mL) exhibit strong *α*-glucosidase inhibition. Likewise, the highest *α-*amylase inhibition was shown by crude extracts of *Ficus religiosa* (IC_50_ 29.2 ± 1.2 *µ*g/mL) and ethyl acetate fractions of *Shorea robusta* (IC_50_ 69.3 ± 1.1 *µ*g/mL), and the highest radical scavenging activity was shown by *F. religiosa* with an IC_50_ 67.4 ± 0.6 *µ*g/mL. Furthermore, to identify the metabolites within the fractions, we employed high-resolution mass spectrometry (LC-HRMS) and annotated 17 known metabolites which justify our assumption on activity. Of 4 medicinal plants examined, ethyl acetate fraction of *S. robusta*, ethyl acetate fraction of *M. melabathrium*, and water or ethyl acetate fraction of *A. catechu* extracts illustrated the best activities. With our study, we set up a foundation that provides authentic evidence to the community for use of these traditional plants. The annotated metabolites in this study support earlier experimental evidence towards the inhibition of enzymes. Further study is necessary to explore the clinical efficacy of these secondary molecules, which might be alternatives for the treatment of diabetes and pathogens.

## 1. Background

Natural products have gained significant appreciation as an alternative and/or complementary healthcare approach with extensive pharmaceutical and biological properties [1, 2]. Worldwide, more than 900 medicinal plants are used for the cure of diabetes by different traditional practices like Ayurveda, Homeopathy, Amchi, Unani, Sidha, Chinese medicine, and folklore [3]. Although a very small number of plants are investigated for the treatment of pathogens, it is found more effective than synthetic drugs [4]. Diversities of secondary metabolites in plants are affected by various factors such as climate, harvest time, storage conditions, genetics, and variability [5].

In Nepal, more than 700 plant species constitute medicinal properties. They are used by different ethnic communities as a local source of medicine mostly called Ayurveda or Homeopathy [6]. As Nepal is climatically diverse, medicinal plants have massive attention among researchers for metabolic enzyme inhibition for the cure of infectious diseases. Proper understanding of traditional knowledge and research over them using more scientific methods might lead to the subsequent treatment of multiple diseases [7]. Diabetes and pathogens are postulated to have a high risk of emergence in Southeast Asia (SEA) [8, 9]. Because of diabetes, 1.2 million people died in SEA in 2019 [8]. The death rate of people dying with infectious diseases is very high [9]. Medicinal plants possess immune-modulatory and antioxidant properties, leading to antibacterial activities. They are known to have versatile immune-modulatory activities by stimulating both nonspecific and specific immunity [10]. Phytochemicals such as vitamins (A, C, E, and K), carotenoids, terpenoids, flavonoids, polyphenols, alkaloids, tannins, saponins, pigments, enzymes, and minerals have hypercholesterolemic, anti-inflammatory, analgesic, antidiabetic, antimicrobial, antiplaque, antihypertensive, and anthelmintic activities [11–14].

The desorption/ionization methods for mass spectrometry had been technologically advanced for chemical identification over the past decades [15]; however, the characterization of plant extract using mass spectrometry is relatively difficult due to the lack of reliable extraction procedures [13, 14, 16]. Quadruple time-of-flight liquid chromatography-mass spectroscopy (Q-TOF LC/MS) technology provides an outstanding separation of major and trivial constituents of natural product extracts along with increasing selectivity and avoiding false-positive findings [17, 18]. This high level of performance allows the elemental composition of each m/z value to be accurately calculated using the software. The high mass resolution of up to 20,000 makes it useful for exact mass measurement as well as for molecular formula generation of any unknown molecule, parent ion, and fragment ion in the plant extracts [19]. This method is rapidly becoming an effective analytical tool to annotate compounds within a short time frame and with high sensitivity. In this study, we prioritized four medicinal plants in Nepal. Along with bioactivity studies, we also characterized/annotated molecules via LC-HRMS. Further purification and characterization of therapeutically novel molecules could be the subject of future work.

## 2. Materials and Methods

### 2.1. Chemicals

The *α*-glucosidase enzyme (CAS No.: 9001-42-7), 4-nitrophenyl *α*-D-glucopyranoside (CAS No.: 3767-28-0), *α*-amylase (CAS No.: 9000-90-2), 2-chloro-4-nitrophenyl-*α*-D-maltotrioside (CAS No.:118291-90-0), acarbose (CAS No.: 56180-94-0), and quercetin (CAS No.: 117-39-5) were purchased from Sigma-Aldrich (Germany). Gallic acid and 2,2-diphenyl-1-picrylhydrazyl (DPPH) were purchased from Molychem (India) and Himedia (India), respectively. Dimethyl sulphoxide, sodium dihydrogen orthophosphate, and other chemicals were purchased from Fisher Scientific (India).

### 2.2. Collection of Plant Materials

Parts of the plants taken under study were collected based on ethnobotanical and traditional medicinal uses of local healers from different areas of Nepal during May-December 2020. Herbarium specimens were deposited at the Central Department of Botany, Tribhuvan University. The list of medicinal plants selected for the study and their ethnopharmacological applications is shown in Table 1. The plants obtained were air-dried in the shade at room temperature and ground into a fine powder.

### 2.3. Preparation of Crude Extracts and Fractionation

The plant materials were shade-dried and ground into a fine powder. Extraction was done by the cold percolation method. Briefly, the powder of different plants was soaked in methanol for 24 hours at room temperature for three successive days. After 24 hours, the dissolved extracts were filtered through the Whatman-1 filter paper and collected and then concentrated at reduced pressure at 50°C using a rotary evaporator to obtain crude extracts. 50 g of the crude extract was then suspended in distilled water and successively partitioned with three different solvents based on their polarity, i.e., n-hexane, followed by dichloromethane and ethyl acetate.

### 2.4. Total Phenolic and Flavonoid Contents

The TPC of the extracts was determined using the Folin-Ciocalteu reagent, as described previously [62, 63] with slight modification. 20 *μ*L of each extract (0.5 mg/mL) was separately mixed with Folin-Ciocalteau's reagent (100 *μ*L, 1 : 10 v/v diluted with distilled water) and aqueous sodium carbonate (Na_2_CO_3_, 80 *μ*L, 1 M) solution, and then the mixture was allowed to stand for 15 minutes at room temperature. The reaction mixture's absorbance was measured at 765 nm using a microplate reader (Synergy LX, BioTek, Instruments, Inc., USA). Gallic acid (GA) was used as a standard, and the total polyphenolic concentration in the extracts was expressed as milligrams of gallic acid equivalent per gram of dry weight (mg GAE/g) of the extract. The assay was performed in triplicate.

The TFC of the extracts was determined using the colorimetric method described previously [64, 65] with slight modification. Briefly, 20 *μ*L of each extract (0.5 mg/mL) was separately mixed with 60 *μ*L ethanol and 5 *μ*L aluminium trichloride (AlCl_3_, 10%). Subsequently, 5 *μ*L of 1 M potassium acetate and 110 *μ*L distilled water were added to each well, and the reaction mixture was allowed to stand for 30 minutes. The absorbance was then measured at 415 nm with a microplate reader against blank. Quercetin was used as standard, and the total flavonoid concentration in the extracts was expressed as milligrams of quercetin equivalent per gram of dry weight (mg QE/g) of extract. For each extract, triplicates of measurements were performed.

### 2.5. Free Radical Scavenging Activity

The free radical scavenging activity was determined using 2,2-diphenyl-1-picrylhydrazyl (DPPH), as described previously [66]. Briefly, 0.1 mM solution of DPPH in methanol was prepared, and an equal volume (100 *μ*L) of this solution was added to an equal volume of plant samples (100 *μ*L) prepared in 30% DMSO at 0.98–500 *μ*g/mL concentrations. It was incubated in the dark for 30 minutes, and then the absorbance was measured at 517 nm. A 100 *μ*L of 0.1 mM DPPH mixed with 100 *μ*L of 30% DMSO (solvent) was used as control. Quercetin was used as the standard reference compound for this assay. The degree of color change from purple (DPPH radical) to yellow (diphenyl picrylhydrazine) obtained after reduction at different concentrations was measured. The capability to scavenge the DPPH radical was calculated by using the following equation:

(1) $$\begin{array}{c} \%\text{ scavenging}=\frac{A_{o}-A_{t}}{A_{o}}\times 100, \end{array}$$

where *Ao* is the absorbance of DPPH with 30% DMSO and *A*_*t*_ is the absorbance of DPPH with a test or reference sample.

### 2.6. *In Vitro α*-Glucosidase and *α*-Amylase Inhibition Assays

The crude extracts for *α*-glucosidase enzyme inhibitory activity were determined by the method described by Fouotsa et al. with slight modification [67]. The *α*-glucosidase (0.2 units) was premixed with 500 *μ*g/mL of extract in 100 mM phosphate buffer saline (pH 6.8). Then, 0.7 mM pNPG in the PBS (phosphate buffer saline) buffer was added as a substrate. This reaction mixture was pre-incubated in a 96-well microplate at 37°C for 15 min. The *α*-glucosidase activity was determined by measuring the p-nitrophenol release from the hydrolysis of pNPG at 405 nm in a microplate with Gene 5 software. Acarbose was used as the standard compound for this assay. The % *α*-glucosidase inhibitory activity was calculated as given in

(2) $$\begin{array}{c} \%\text{ inhibition}=\frac{A_{o}-A_{t}}{A_{o}}\times 100, \end{array}$$

where *A*_*o*_ is the absorbance of enzyme-substrate reaction with 30% DMSO and *A*_*t*_ is the absorbance of enzyme-substrate with plant extract.

*α*-Amylase inhibition assay was carried out as described in our previous study [68]. Briefly, 20 *µ*L of plant extracts and 80 *µ*L of porcine pancreas *α*-amylase (1.5 U/mL) in phosphate buffer of pH 7.0 were loaded in microplate wells, and initial absorbance was taken. The plate was incubated at 37°C for 15 minutes. Then, 100 *µ*L of a substrate (0.5 mM), CNPG3 (in the same buffer), was added to start the reaction with incubation for 15 minutes, and change in absorbance was monitored at 405 nm [68]. The DMSO was taken as control. All the experiments were performed in triplicate in a final volume of 200 *μ*L by using a microplate reader. The % *α*-amylase inhibitory activity was calculated using equation (2), mentioned earlier.

### 2.7. Antimicrobial Assay of Plant Extracts

Antimicrobial assay of extracts was performed by the agar well diffusion method in Mueller Hinton Agar (MHA) plates [69]. The test organisms were inoculated in Muller Hinton Broth (MHB) and incubated at 37°C to adjust the turbidity to 0.5 McFarland standards giving a final inoculum of 1.5 × 10^8^ CFU/mL. The MHA plates were lawn-cultured with the above maintained microbial inoculum. Plant extracts of 50 mg/mL concentration were prepared in 50% DMSO. Six wells of 6 mm were bored in the cultured lawn media with the help of a sterile cork-borer (6 mm). Each well was filled with 50 *μ*L plant extract with the positive control (neomycin 50 mg/mL) and negative control (50% DMSO) in each experiment set. It was allowed to diffuse for about 15 minutes at room temperature and incubated for 18–24 hours at 37°C. After incubation, plates were observed to form a clear, i.e., zone of inhibition (ZoI) around the well and measured in mm.

### 2.8. Determination of MIC and MBC

The broth microdilution method was used to determine the MIC and MBC, according to Clinical and Laboratory Standards Institute (CLSI) [70]. Twofold serial dilutions of extracts were prepared directly in sterile 96-well microdilution plates with flat bottom wells containing MHB to obtain various concentrations. The bacterial inoculum was added at a final concentration of 10^6^ CFU/mL by diluting 1 : 100 the 0.5 McFarland turbidity culture in MHB. Finally, five *μ*L of bacteria was added to each well except for negative control.

Neomycin is a standard drug that was used as a positive control. The plate was covered with a sterile lid and incubated for 24 h at 37°C. Resazurin (0.003%) was added to each well of the microtiter plate and was incubated at 37°C for 3–4 hrs. The wells containing the bacterial growth turned into pink color, whereas the well without bacterial growth remained blue. The MIC was considered as the lowest concentration of the extract that completely inhibits bacterial growth. The MBC was determined by streaking the content of wells onto NA plates with incubation of over 18 hours at 37°C [70].

### 2.9. Molecular Annotation

Plant fractions were subjected to LC-HRMS. Identification of metabolites from an active fraction of water and ethyl-acetate extract of *A. catechu,* ethyl acetate fraction of *S. robusta,* ethyl acetate fraction of *M. melabathrium,* and hexane fraction of *F. religiosa* was carried out at Sophisticated Analytical Instrument Facility (SAIF), CSIR-Central Drug Research Institute, Lucknow. Samples were analyzed on an Agilent 6520, Accurate-Mass Q-TOF Mass Spectrometer equipped with a G1311A quaternary pump, G1329A autosampler, and G1315D diode array detector (DAD). The solvent system consists of acetonitrile (ACN) and 5mM acetate buffer and water at the flow rate of 0.5 ml/min. The initial condition started from 5% ACN for 0.1 min to 30% ACN for 6 min, 80% ACN for 20 min, and back to its initial conditions. During the whole process, column temperature was maintained at 30°C. After passing through the DAD flow cell, the column elute was directed to Q-TOF HRMS fitted with an electrospray interface (ESI). The MS analysis was carried out using an ESI-positive ionization mode with mass ranges from 100–3000 Da. Raw data obtained from the LC/HRMS system were converted to mzML format using the ProteoWizard tool MSConvertGUI [71]. MZmine 2 was used for peak detection, peak alignment, and identification (target compound annotation) using centroid data [71–73].

### 2.10. Data Analysis

The results were processed by using Gen5 Microplate Data Collection and Analysis Software and then by MS Excel. The IC_50_ (inhibition of enzymatic hydrolysis of the substrate pNPG and CNPG3 by 50%) value was calculated using the Graphpad Prism software version 8. Values were expressed as a mean ± standard error of the mean of triplicate. The LC-HRMS metabolic was processed in an untargeted manner with MZmine 2 and used MestreNova 12.0 tool to annotate the molecules. The metabolites were searched using the Pubchem database, and Dictionary of Natural Products 2.

## 3. Results and Discussion

### 3.1. Total Phenolic and Flavonoid Contents

The TPC and TFC were expressed as the GAE/g, and QE/g of extract using a calibration curve of gallic acid and quercetin, respectively. The TPC of plant extracts follows the order *A. catechu* > *S. robusta* > *M. malabathricum* > *F. religiosa.* Likewise, the TFC of plant extracts follows the order *S. robusta* > *M. malabathricum* > *F. religiosa* *>* *A. catechu*, which are shown in Table 1S.

### 3.2. Free Radical Scavenging Activity

In this study, the antioxidant activities of crude extracts of plants are shown in Table 2S. DPPH radical scavenging results were reported as IC_50_ and compared with the IC_50_ value of quercetin (6.3 ± 1.0 *µ*g/mL) as a standard. The radical scavenging ability of plant extracts follows the order *F. religiosa* (67.4 ± 0.6 *µ*g/mL) > *M. malabathricum* (74.9 ± 5.6 *µ*g/mL) > *A. catechu* (84.9 ± 1.9 *µ*g/mL) > *S. robusta* (111.4 ± 1.1 *µ*g/mL). The antioxidant ability is shown by providing hydrogen or electrons from the phytochemicals within extracts to free radicals.

### 3.3. *α*-Glucosidase Inhibitory Activity

The results of *α*-glucosidase inhibitory activity of the extracts are given in Table 2. Among the tested fraction, ethyl acetate and water fraction showed the most potent activity with an IC_50_ value ranging within 9–114.9 *µ*g/mL against the *α*-glucosidase enzyme as compared to acarbose (IC_50_ = 344.23 ± 1.03 *µ*g/mL). The ethyl acetate fraction of plants understudy on *α*-glucosidase inhibitory activity follows the order as *M. malabathricum* > *S. robusta* > *F. religiosa* > *A. catechu* while that of aqueous fraction follows *A. catechu* > *M. malabathricum* > *S. robusta* > *F. religiosa*.

### 3.4. *α*-Amylase Inhibitory Activity

In the *α*-amylase assays (Table 2), crude methanol extracts and their ethyl acetate fractions effectively inhibited the enzyme activity compared to other fractions. Crude extracts of *F. religiosa* followed by that of *S. robusta* and *A. catechu* showed antidiabetic activity with an IC_50_ of 29.24 ± 1.173, 89.127 ± 0.758, and 115 ± 4.004 *µ*g/mL, respectively, compared to acarbose (IC_50_ = 6.1 ± 0.1 *µ*g/mL). The hexane fraction of *F. religiosa* bark extract inhibited *α*-amylase with IC_50_ of 68.234 ± 5.812 *µ*g/mL. Only the aqueous fraction of *A. catechu* was significant towards *α*-amylase.

### 3.5. Antimicrobial Activity

**Figure 1:**
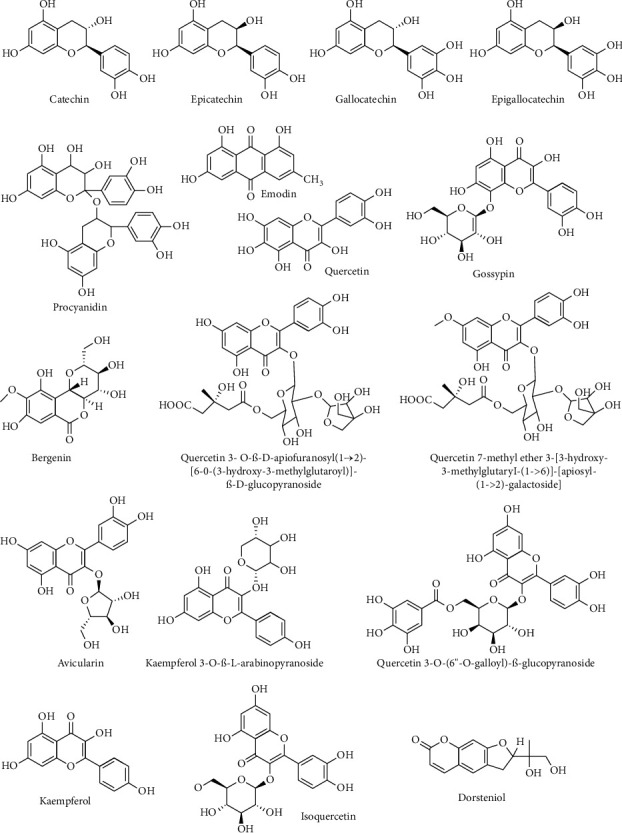
Secondary metabolites annotated during the study.

Antimicrobial activity of different fractions of plants was tested against ATCC strains such as *Staphylococcus aureus* ATCC 43300, *Escherichia coli* ATCC 2591, *Klebsiella pneumoniae* ATCC 700603, *Salmonella typhi* ATCC 14028, and *Shigella sonnei* ATCC 25931. The ZoI of each fraction of plants is shown in Table 3. The representative pictures showing ZoI of *A. catechu* extract against *S. aureus* ATCC 43300 and *E. coli* ATCC 2591 are shown in Figure 1S.

### 3.6. Minimum Inhibitory and Bactericidal Concentration

Different fractions were examined for MIC and MBC against five ATCC strains such as *S. aureus* ATCC 43300*, E. coli* ATCC 25931, *K. pneumonia* ATCC 700603, *S. typhi* ATCC 14028, and *S. sonnei* ATCC 25931. The MIC of different fractions against ATCC reference strains was found between 0.3 mg/mL and 25 mg/mL shown in Table 4. *M. malabathricum* and *S. robusta* extract showed inhibition at lower concentrations, but it requires more concentration to kill the bacterial growth, as evidence by MIC and MBC values. MIC of water fraction of *A. catechu* and ethyl acetate fraction of *F. religiosa* against *K. pneumonia* ATCC 700603 is shown in Figure 2S. Likewise, MBC of ethyl acetate fraction of *A. catechu* and *M. malabathricum* against *S. sonnei* ATCC 25931 is shown in Figure 3S.

### 3.7. Molecular Annotation

The raw data of LC-HRMS were processed using MZmine and MestreNova 12.0 tool was used for further analysis. The total ion chromatogram (TIC) of the fractions subjected for LC-HRMS analysis is shown in Figure 4S. These results were verified with available public compound databases. Compounds were annotated from the fractions of all 4 plants. The annotated molecules from our spectra are catechin (Figure 5S), epicatechin (Figure 5S), gallocatechin (Figure 6S), epigallocatechin (Figure 6S), procyanidin (Figure 7S), emodin (Figure 8S), quercetin (Figure 9S), gossypin (Figure 10S), bergenin (Figure 11S), quercetin 3- O-*β*-D-apiofuranosyl(1 ⟶ 2)-[6-O-(3-hydroxy-3-methylglutaroyl)]-*β*-D-glucopyranoside (Figure 12S), quercetin 7-methyl ether 3-[3-hydroxy-3-methylglutaryl-(1->6)]-[apiosyl-(1->2)-galactoside] (Figure 13S), avicularin (quercetin 3-*α* -L-arabinofuranoside) (Figure 14S), kaempferol 3-O-*α*-L-arabinopyranoside (Figure 15S), quercetin 3-O-(6”-O-galloyl)-*β*-glucopyranoside (Figure 16S), kaempferol (Figure 17S), isoquercetin (Figure 18S), and dorsteniol (Figure 19S) which are shown in Table 5 (Figure 1).

## 4. Discussion

Plants synthesize a wide range of secondary metabolites which showed broad-spectrum enzyme inhibitory potential. Nepal is a Himalayan nation with lopsidedly rich flora and fauna located at a transition zone between the flora of the Western Himalaya and the Eastern Himalaya [74]. The assorted variety of species in Nepalese flora offers incredible open doors for the hunt of medicinal substances. Nepal has been underexplored in terms of medicinal plants. Due to poor infrastructures and funding in research, most of the analytics have not been explored out of its biodiverse flora and fauna. Though there are some works in the past, most of them are restricted only up to the crude activity. No research group has gone so far as to annotate compounds from Nepalese plants. Khadayat et al. studied the *α*-amylase inhibitory activity of Nepalese medicinal plants up to crude extract. Aryal et al. and Phuyal et.al. studied only TPC, TFC, and antioxidant potential of plants available in Nepal [68, 75, 76]; Sai et al. and Subba et al. studied phytochemical screening, antioxidant and *α*-amylase inhibitory activities of medicinal plants from Nepal [77, 78]. Since natural products have contributed 48.64% as a source of new drugs for diabetes [79], we aim to annotate secondary metabolites from Nepalese plants covered by multiple pharmacological applications based on previous experiments.

Compounds like quercetin, ferulic acid, anthocyanins, catechin, and resveratrol, which are plant-based phenolics and flavonoids, are responsible for regulating glycemia through insulin secretion, increased glucose uptake, lipid peroxidation inhibition, and inhibition of enzymes like *α*-glucosidase, and *α*-amylase [80, 81]. Due to the vast health benefits such as antioxidant, antimicrobial, antiulcer, antidiabetic, hepatoprotective, and anticarcinogenic activities of phenolic and flavonoid compounds, thus they are considered as a major class of phytochemicals [82, 83].

Previously, evidence of antidiabetic activity of *M. malabathricum* leaves extract has been shown by *in vivo* experiments on diabetes-induced rats [47, 84] to support our results. *A. catechu* (L.f.) Willd. leaf extracts showed an IC_50_ of 0.4977 mg/mL against *α*-glucosidase [85]. Similarly, kaempferol, quercetin, naringenin, and baicalein were the flavonoids isolated from *F. racemosa* stem bark showing a significant decrease in blood glucose levels in diabetes-induced albino Wistar rats [86]. Additionally, gossypetin, herbacetin, kaempferol, leucoperalgonidin, leucodelphinidin, and sorbifolin from *Ficus* species were shown as potential digestive enzymes inhibitors through in silico analysis [87].

Phenolic and flavonoid compounds play a major role in free radical scavenging activity via chain termination of free radicals that depend on hydroxyl groups' number and position by donating hydrogen atoms [88]. The structural features like many hydroxyl groups and their configuration, the ketonic functional group at C-4, and a double bond at C2–C3 on flavonoids enhanced the antioxidant ability [89, 90]. Due to these structural features, extracts with higher phenolic compounds are already proved to have a higher antidiabetic ability via the formation of hydrogen bonds and hydrophobic interactions with enzymes and reduce their activity [91, 92]. There are high correlations between antioxidant capacity and total phenols and total flavonoids, so the presence of a high concentration of phenolic compounds also has high antioxidant activity [75]. There is no correlation between high TPC and TFC value with high antioxidant activity in our study. Bioactive compounds present in plant extracts showed antidiabetic activity by inhibiting glucose absorption in the intestinal tract (inhibiting the different steps of carbohydrates digestion) or antioxidant ability [93].

Phytochemicals such as vitamins (A, C, E, and K), carotenoids, terpenoids, flavonoids, polyphenols, alkaloids, tannins, saponins, pigments, enzymes, and minerals have antimicrobial and antioxidant activity [13, 14]. The antimicrobial activity of extracts from different parts of *A. catechu* [22, 94–97], *F. religiosa* [98], *M. malabathricum* [55, 99], and *S. robusta* [100] has been shown previously. However, the variation in activity is mainly due to diversities in phytochemicals. *A. catechu* extracts inhibit the growth of *Bacillus subtilis*, *S. aureus*, *S. typhimurium E. coli*, and *Pseudomonas aeruginosa* with MIC of 1,000 *µ*g/mL against *S. aureus* and *B. subtilis* while that for *S. typhimurium, E. coli*, and *P. aeruginosa* was 700, 1,500, and ≤2,000 *µ*g/mL, respectively [22]. *M. malabathricum* has been shown with activity against *S. aureus, B. subtilis, B. cereus, E. coli, P. aeruginosa, S. typhi,* and *K. pneumonia* [55]. Ismail showed extracts from *Melastoma* species exhibited MIC activity ranges from 12.5 to 100 mg/mL against *E. coli, P. Aeruginosa, B. cereus,* and *S. aureus* [101]. Antimicrobial activity of *S. robusta* against *P. aeruginosa, S. aureus, E. coli, E. faecalis, C. albicans,* and *A. niger* showed ZoI of 7–25 mm [100].

Catechin, epicatechin, and taxifolin from *A. catechu* [26, 96]; ursolic acid, 2*α*-hydroxyursolic acid, asiatic acid, *β*-sitosterol 3-*O*-*β*-*D*-glucopyranoside, quercetin, ellagic acid, and kaempferol from *M. malabathricum* [55]; and amarogentin, amaroswerin, gentianine, mangiferin, oleanolic acid, swechirin, swertiamarin, sweroside, swertanoone, ursolic acid, etc. from *S. chirayita* [102, 103] were identified with antibacterial ability.

The mechanism behind the antimicrobial activity of phenolic compounds and flavonoids is the inhibition of nucleic acid synthesis, cytoplasmic membrane function, energy metabolism, attachment and biofilm formation, and porin on the cell membrane, and membrane permeability alteration leading to cell destruction as well as attenuation of pathogenicity [104]. In our study, most of the extracts showed higher antibacterial activity against gram-positive bacteria as compared to gram-negative bacteria; it might be due to differences in the cell wall structure as the peptidoglycan layer in gram-positive bacteria is an ineffective permeability barrier as compared to gram-negative bacteria containing outer membrane and a unique periplasmic space rich in lipopolysaccharide molecules, which act as a barrier for the penetration of numerous antimicrobial compounds [105].

The most time-consuming steps are metabolite identification. Because of the time-consuming and the availability of plant materials, we performed LC/HRMS for metabolite profiling based on *in vitro* assay rather than performing high-performance liquid chromatography (HPLC), LC-HRMS/MS, and nuclear magnetic resonance (NMR). Likewise, after performing LC-HRMS we proceeded with our raw data with MZmine and overlayed our ion chromatogram. *A. catechu* may contain catechin or epicatechin: base peak m/z 291.086 (elemental composition C_15_H_14_O_6_; t_R_ 6.25); fragments peak at 139 and [M + Na]^+^ peak at m/z 313.2 comparing our result with Hye et al., Ibrahim et al., Shen et al., and Wang et al., [106–109]. The fragmentation pattern of catechin/epicatechin is shown in Figure 20S. It is predicted to be gallocatechin/epigallocatechin and procyanidin: in our spectra, we observed [M + H]^+^ peak m/z 307.08 (C_15_H_14_O_7_;* t*_R_ 5.22); fragment peak at m/z 289 and 139 for gallocatechin/epigallocatechin. The fragmentation pattern of gallocatechin/epigallocatechin is shown in Figure 21S. Likewise, the molecular ion peak at m/z 579.15 (C_30_H_26_O_12_; *t*_R_ 5.79) with catechin-like RDA fragment [M + H − 152]+ at m/z 427.13 is speculated as procyanidin similar to the study by Shen et al. [108]. Additionally, the quasimolecular ion peak at m/z 271.06 (C_15_H_10_O_5_; *t*_R_ 18.65) with fragment peak at m/z 253, 243, 229, 225, and 197 is annotated as emodin consistent with the study by Zhan et al. The fragmentation pattern of emodin is shown in Figure 22S.

The absence of myricetin and the presence of proanthocyanidin in the leaves of *S. robusta* were very significant [111, 112]. Base peak with m/z 303.051 (C_15_H_10_O_7_; *t*_R_ 8.04) was expected to be quercetin. The fragmentation pattern of the quercetin is shown in Figure 23S. Similarly, base peak with m/z 481.09 (C_21_H_20_O_20_; *t*_R_ 6.82) and the fragment ion at m/z = 319.05 and 287.15 is interpreted as gossypin comparing our spectra with Giorio et al. and Petsalo et al. [113, 114]. The base peak at m/z 329.08 (molecular formula C_14_H_16_O_9;_* t*_R_ 5.11) and quasi molecular ion peak [M + Na]^+^ at m/z 351.06 are expected to be bergenin isolated from *S. robusta* with reference from the data collected from our spectra and Mukherjee et al. [60]. The other flavonoid is expected to be quercetin 3- O-*β*-D-apiofuranosyl(1 ⟶ 2)-[6-O-(3-hydroxy-3-methylglutaroyl)]-*β*-D-glucopyranoside: base peak m/z 741.18 (C_32_H_36_O_20_; *t*_R_ 11.52), [M + Na]^+^ m/z = 763.16 and the fragment ion at m/z = 609.16 [M + H − 132 (pentose)]^+^ with reference from the data collected from our spectra and Fu et al., 2010 [115]. Adding 14 Da mass on quercetin 3-O-*β*-D-xylopyranosyl(1 ⟶ 2)-[6-O-(3-hydroxy-3-methylglutaroyl)]-*β*-D-glucopyranoside yields mass equals 755 which could be quercetin 7-methyl ether 3-[3-hydroxy-3-methylglutaryl-(1->6)]-[apiosyl-(1->2)-galactoside] [116]. Likewise, the base peak with m/z 435.09 (C_20_H_18_O_11_; *t*_R_ 7.83) and fragments peak at m/z 303.05, 287.05, 183.14, and 153.02 are predicted to be avicularin (quercetin 3-*α* -L-arabinofuranoside) as spectral figures match with the literature of Santos et al. [117]. Moreover, in our spectra, the base peak with m/z 419.09 (C_20_H_18_O_10_; *t*_R_ 8.22) and fragments peak at m/z 287.09 ([(M + H) − 132]) are annotated as kaempferol 3-O-*α*-L-arabinopyranoside taking reference of Vuković et al. [118].

*M. melabathrium* may contain isoquercetin since in our spectra at base peak m/z 465.10 (*t*_R_ 7.58) and fragments ions at m/z 303.04 (quercetin) and 289.06 (kaempferol) exactly match with the spectra data of Liu et al. [119]. Base peak at m/z 617.11 (*t*_R_ 7.16) and fragments ions at m/z 303.05 corresponding to [aglycone + H]^+^ and at m/z 315.07 corresponding to [sugar + gallic acid + H]^+^ are assumed to be quercetin 3-O-(6”-O-galloyl)-*β*-glucopyranoside taking reference of Şöhretoğlu et al. [120]. The other metabolite was reported as kaempferol with base peak m/z 287.05 (C_15_H_10_O_6_; *t*_R_ 8.69) and fragments ion at m/z 259.12, 165.05, 153.0, 127.03, and 121.09 respectively. The spectra are similar to March et al. [121]. The fragmentation pattern of kaempferol is shown in Figure 24S. Similarly, from MZmine total ion chromatogram (TIC) we can say that the *M. melabathrium and S. robusta* may contain quercetin at base peak m/z 303 as we can observe the peak of quercetin coinciding in both plants.

In hexane fraction of *F. religiosa*, we did defatting by removing the first hexane fraction. But the chromatogram was also seen in our mass spectrometry with fairly fewer amounts. Then, we annotated dorsteniol secondary metabolites in *F. religiosa*. Literature search shows that *Ficus* species contain dorsteniol. Our spectra data shows base peak at m/z 263.09 (C_14_H_14_O_5_; *t*_R_ 8.14) and [M + Na]^+^ peak at m/z 285.07 as in Alqasoumi et al. [122]. So, the predicted compound is dorsteniol.

In our study, we have annotated 17 secondary metabolites from different plant extract. To support our annotation, more spectroscopic data are required. Crude extracts showed potent activity due to the synergistic effect of compounds. Besides that, ethyl acetate and water fraction showed potent activity; this might be due to phenolic and flavonoid compounds present in those solvents. In some extracts, hexane and DCM showed good inhibition due to terpenes, steroids, and diterpenes present in extracts.

## 5. Conclusions

Due to the rising prevalence of diabetes and antimicrobial resistance, the quest for novel drugs from natural sources persists. The scientific study of different traditional medicinal plants used by diverse groups of people is necessary. Traditional medicinal plants that local peoples have used were screened for antioxidant, antimicrobial, and antidiabetic assay. Among 4 selected medicinal plants from Nepal, most extracts and their fractions can show antioxidant, antimicrobial, and antidiabetic activity, supporting these plants' traditional use. Further works can be carried out on isolation, identification, in silico, and kinetics of potential inhibitors from an active fraction of plant extracts used as a drug candidate for diabetes.

## Figures and Tables

**Table 1:** List of medicinal plants selected for the study and their ethnopharmacological applications.

Scientific name	Family	Voucher specimen	Indigenous uses	Chemical constituents
*A. catechu* (L.F.) wild	Fabaceae	TUCH-201011	It is used for anticancer purposes. It is useful in cold and cough, ulcers, boils, and eruptions of the skin, bleeding piles, antipyretic, acute, and chronic wound healing [20–23]. Catechin and taxifolin possess antifungal, antiviral, antibacterial, anti-inflammatory, and antioxidant activity [22].	4-Hydroxybenzoic acid, kaempferol, quercetin, 3,4′,7-trihydroxyl-3′,5-dimethoxyflavone, catechin, epicatechin, afzelechin, epiafzelechin, mesquitol, ophioglonin, aromadendrin, phenol [23]; rhamnetin, 4-hydroxyphenol, 3,3′,5,5′,7-pentahydroxyflavane, fisetinidol, 5-hydroxy-2-[2-(4-hydroxyphenyl)acetyl]-3-methoxybenzoic acid,n(2s,3S)-3,7,8,3′,4′-pentahydroxyflavane [24]; rutin [25]; taxifolin [26]; quercetin 3-methyl ether, caryatin, ellagic acid [27]; robinetinidol [28]; gallochin, gossypetin, phlobatannin, quercitin [29]
*F. religiosa*	Moraceae	TUCH-201014	Bark is used in diarrhoea, dysentery, anti-inflammatory, antibacterial, cooling, astringent, gonorrhoea, and burns [30, 31]. Leaves are used in hiccups, vomiting, cooling, gonorrhoea, asthma, cough, diarrhoea, and gastric problems. Similarly, fruits are used in fever, tuberculosis, paralysis, asthma, and digestion [31–33]. It is used for anticancer activity [34], antioxidant, wound healing and anti- inflammatory activity [35], antidiabetic activity [36], and antiamnesic activity [37]. Lupeol possesses anti-inflammatory activity [38]. Quercetin and kaempferol possess antifungal property [39, 40].	Naphthyl-1,3-diol-1-(3*β*-lanost-5,24-dienyl)-3-n-octadec- 9,12,15-trienoate, naphthyl-1,3-diol-1-(3*β*-lanostan-19-oic acid-yl)-3- n-octadec-9,12-dienoate, triterpenic ester lanostan-19-oic acid-3*β*-olyl-n-octadec-9,12,15-trienoate, *β*-sitosteryl oleate, *β*-sitosterol glucoside [41]; lupenol, *γ*-sitosterol, 1,2-Benzenediol [42]; phenol, salicylaldehyde, phenylacetaldehyde, allyl caproate, linalool, n-nonanal, adipoin, methylcyclopentane, 2-dione, itaconic anhydride, 2-phenylethyl alcohol, benzeneacetonitrile, nonadienal, nonen-1-ol, nonadienol, linalool oxide, catechol, coumaran, cinnamyl alcohol, vinylguaiacol, hexenyl tiglate, eugenol, hexenyl hexenoate, *β*-ionone, dihydroactinidiolide, *α*-copaene, hexenyl benzoate, eudesmol, eudesmol, epi-*α*-cadinol, *β*-eudesmol, *α*-eudesmol, *α*-cadinol, pentadecanal, palmitic acid and itaconic anhydride, 3-methylcyclopentane-1, 2-dione [43]; undecane, tridecane, tetradecane, (e)-*β*-ocimene *β*-bourbonene, *β*-caryophyllene, *α*-trans bergamotene, *α*-thujene, *α*-pinene, *β*-pinene, *α*-terpinene, limonene, dendrolasine, dendrolasine *α*-ylangene, *α*-copaene, aromadendrene, *α*-humulene, alloaromadendrene, germacrene, bicycle-germacrene, *γ*-cadinene and *δ*-cadinene [44, 45]
*M. melabathrium*	Melastomataceae	TUCH-201013	It is used in looseness of the bowels, the runs, hemorrhoids, leucorrhoea, wounds and cut lightening urinary issues, leucorrhea, urinary tract, and toothache [46, 47]; antiviral activity, cytotoxicity activity [48], wound healing activity [49], antiulcer activity, antivenom activity [50], antidiabetic activity, antioxidant activity, antihyperlipidemic activity [47]. *β*-Sitosterol has shown antidiabetic activity [51]. Betulinic acid, quercetin, quercitrin, *α*-amyrin show anti-inflammatory activity [52].	*β*-Sitosterol, *β*-sitosterol-3-O-*β*-D-glucopyranoside, ursolic acid, asiatic acid, 2-hydroxyursolic acid, Kaempferol [53]; *α*-amyrin, betulinic acid, kaempferol-3-O-*β*-D-glucoside [54]; 2*α*-hydroxyursolic acid, *β*-sitosterol 3-O-*β*-D-glucopyranoside, 1,2-dilinolenyl-3-O-*β*-D-galactopyanoside, kaempferol, kaempferol 3-*O*-*α*-*L*-rhamnopyranoside, kaempferol 3-*O*-*β*-*D*-glucopyranoside, kaempferol 3-*O*-*β*-*D*-galactopyranoside, kaempferol 3-O-(2″,6″-di-O-E-p-coumaryl)-*β*-D-galactopyranoside, quercetin, ellagic acid [55]; gallic acid, benzoic acid, epicatechin, malabathrin, casuarictin [56]
*S. robusta* gaertn	Dipterocarpaceae	TUCH-201010	It is used in the treatment of ulcer, cough, itching, leprosy, and as anthelmintic [57]. Antibacterial wound healing and anti-inflammatory activity due to the presence of polyphenols, flavonoids, and triterpenoids, etc. Ursolic acid, amyrin extracted from this plant is responsible for showing antibacterial activity and antiulcer [58].	Ammarenolic acid, asiatic acid [59]; ursolic acid, tri and tetrahydroxy ursenoic acid, *α* and *β*-amyrin, *α* –amyrenone, mangiferonic acid, benthamic acid and uvaol [58]; bergenin [60]; caryophyllene oxide, calarene epoxide, lupeol, *β*-humulene, *α*-amyrin, *β*-caryophyllene [61]

**Table 2:** *α*-Glucosidase and *α*-amylase inhibitory activity and IC_50_ (*µ*g/mL) values of various fractions.

Plants	Crude extract		Hexane fraction		DCM fraction		Ethyl acetate fraction		Water fraction	
	G	A	G	A	G	A	G	A	G	A
*A. catechu*	23.7 ± 0.7	115 ± 4.0	<50%	<50%	75.1 ± 3.6	<50%	57.6 ± 1.9	<50%	9.0 ± 0.6	73.2 ± 4.3
*F. religiosa*	28.5 ± 0.7	29.2 ± 1.2	18.2 ± 0.2	70.2 ± 5.8	<50%	<50%	24.7 ± 0.1	78.93 ± 1.1	114.9 ± 2.7	<50%
*M. malabathricum*	82.6 ± 3.0	221.2 ± 1.9	<50%	<50%	<50%	<50%	9.1 ± 0.3	213.5 ± 2.4	11.6 ± 0.6	<50%
*S. robusta*	34.8 ± 0.7	89.1 ± 0.8	188.5 ± 2.4	<50%	200.8 ± 4.6	<50%	21.4 ± 0.5	69.3 ± 1.1	50.9 ± 1.5	<50%

G: *α*-glucosidase and A: *α*-amylase.

**Table 3:** Zone of inhibition (mm) of each fraction of plants.

	*Acacia catechu* (fraction)				*Shorea robusta* (fraction)				*Melastoma malabathricum* (fraction)				*Ficus religiosa* (fraction)				PC
	H	D	E	W	H	D	E	W	H	D	E	W	H	D	E	W	
*S. aureus*	10	9	12	14	9	—	—	—	10	—	—	—	10	—	—	—	10
*E. coli*	—	—	—	—	—	—	—	—	—	—	—	—	—	—	7	—	16
*K. pneumoniae*	9	6	10	13	—	7	—	8	—	—	—	—	8	—	10	8	19
*S. typhi*	—	—	—	—	—	—	—	—	—	—	—	—	—	—	—	—	18
*S. soni*	6	9	11	10	—	8	14	15	—	8	16	15	7	—	10	—	25

H: hexane; D: DCM; E: ethyl acetate; W: water; PC: positive control (neomycin).

**Table 4:** Minimum inhibitory concentrations and minimum bactericidal concentrations (mg/mL) of different fractions against microorganisms.

	*Acacia cathecu*		*Shorea robusta*		*Melostoma malabathricum*		*Ficus religiosa*		Positive control	
	MIC	MBC	MIC	MBC	MIC	MBC	MIC	MBC	MIC	MBC
*S. aureus*	3.1	25	0.3	6.25	3.1	25	3.1	12.5	0.062	0.25
*E. coli*	—	—	—	—	—	—	12.5	50	0.004	0.008
*K. pneumoniae*	12.5	25	—	—	—	—	6.25	25	0.004	0.008
*S. typhi*	—	—	—	—	—	—			0.004	0.03
*S. sonnei*	6.25	12.5	6.25	25	1.56	25	3.12	12.5	0.002	0.06

**Table 5:** Secondary metabolites from the different fractions of plants.

Annotated compounds	Calculated Mass	Observed Mass	Formula	Double bond equivalence	Absolute error (ppm)	Retention time (*t*_R_) minute	Source
Catechin or epicatechin	290.07	291.08	C_15_H_14_O_6_	9	0.16	6.25	*A. catechu* (W and E)
Gallocatechin or epigallocatechin	306.07	307.08	C_15_H_14_O_7_	9	0.65	5.22	*A. catechu* (W and E)
Procyanidin	578.15	579.15	C_30_H_26_O_12_	18	0.79	5.87	*A. catechu* (W and E)
Emodin	270.05	271.06	C_15_H_10_O_5_	11	2.8	18.65	*A. catechu* (E)
Quercetin	302.04	303.05	C_15_H_10_O_7_	11	3.65	8.04	*S. robusta/A. catechu/M. malabathricum* (E)
Gossypin	480.09	481.09	C_21_H_20_O_13_	12	1.84	8.8	*S. robusta* (E)
Bergenin	328.08	329.08	C_14_H_16_O_9_	7	2.91	5.11	*S. robusta* (E)
Quercetin 3- O-*β*-D-apiofuranosyl(1 ⟶ 2)-[6-O-(3-hydroxy-3-methylglutaroyl)]-*β-*D-glucopyranoside	740.18	741.18	C_32_H_36_O_20_	15	4.31	11.52	*S. robusta* (E)
Quercetin 7-methyl ether 3-[3-hydroxy-3-methylglutaryl-(1->6)]-[apiosyl-(1->2)-galactoside]	754.19	755.19	C_33_H_38_O_20_	15	—	12.76	*S. robusta* (E)
Avicularin (quercetin 3-*α* -L-arabinofuranoside)	435.09	434.08	C_20_H_18_O_11_	12	3.9	7.83	*S. robusta* (E)
Kaempferol 3-O-*α*-L-arabinopyranoside	418.08	419.08	C_20_H_18_O_10_	12	2.83	8.22	*S. robusta* (E)
Quercetin 3-O-(6”-O-galloyl)-*β*-glucopyranoside	616.1	617.11	C_28_H_24_O_16_	17	0.65	7.16	*M. malabathricum* (E)
Kaempferol	286.04	287.05	C_15_H_10_O_6_	11	0.19	8.69	*M. malabathricum* (E)
Isoquercetin	464.09	465.1	C_21_H_20_O_12_	12	0.43	7.58	*M. malabathricum* (E)
Dorsteniol	262.08	263.09	C_14_H_14_O_5_	8	1.19	8.14	*F. religosa* (H)

E: ethyl acetate; W: water; H: hexane.

## Data Availability

The data used to support the findings of this study are available from the corresponding author upon request.

## Abbreviations

DPPH: 2,2-Diphenyl-1-picrylhydrazyl
DM: Diabetes mellitus
CNPG3: 2-Chloro-4-nitrophenyl-*α*-D-maltotrioside
IDF: International Diabetes Federation
LC-HRMS: Liquid chromatography-high resolution mass spectrometry;
MHB: Mueller Hinton Broth
MIC: Minimum inhibitory concentration
MBC: Minimum bactericidal concentration
PNPG: 4-Nitrophenyl-*α*-D-glucopyranoside
*t*_R_: Retention time
ZoI: Zone of inhibition
MDR: Multidrug resistance
SEA: Southeast Asia
CLSI: Clinical and laboratory standards institute
TIC: Total ion chromatogram.

## Supplementary Materials

Supplementary Materials Table 1S: Total phenolic and flavonoids content of different plants. Table 2S: Antioxidant ability of plant extracts. Figure 1S: Antimicrobial activity of *A. catechu* extracts against *S. aureus* ATCC 43300 and *E. coli* ATCC 2591. Figure 2S: MIC of ethyl acetate of *A. catechu* and *F. religiosa* against *S. sonnei* ATCC 25931. Figure 3S: MBC of ethyl acetate fraction of *A. catechu* and *M. malabathricum* against *S. sonnei* ATCC 25931. Figure 4S: Total ion chromatogram (TIC) of (a) dark blue line: *A. catechu* ethyl acetate fraction, (b) red line: *S. robusta* ethyl acetate fraction, (c) green line: *A. catechu* water fraction, (d) pink line: *M. malabathricum* ethyl acetate fraction, and (e) sky blue line: *F. religiosa* hexane fraction. Figure 5S: Mass spectrum of catechin or epicatechin from *A. catechu*. Figure 6S: Mass spectrum of gallocatechin or epigallocatechin from *A. catechu*. Figure 7: mass spectrum of procyanidin from *A. catechu*. Figure 8S: Mass spectrum of emodin from *A. catechu*. Figure 9S: mass spectrum of quercetin from *S. robusta*. Figure 10S: Mass spectrum of gossypin from *S. robusta*. Figure 11S: Mass spectrum of bergenin acid from *S. robusta*. Figure 12S: Mass spectrum of quercetin 3-O-*β*-D-apiofuranosyl (1 ⟶ 2)-[6-O-(3-hydroxy-3-methylglutaroyl)]-*β*-D-glucopyranoside from *S. robusta*. Figure 13S: Mass spectrum of quercetin 7-methyl ether 3-[3-hydroxy-3-methylglutaryl-(1->6)]-[apiosyl-(1->2)-galactoside] from *S. robusta*. Figure 14S:Mass spectrum of avicularin (quercetin 3-*α* -L-arabinofuranoside) from *S. robusta*. Figure 15S: Mass spectrum of kaempferol 3-O-*α*-L-arabinopyranoside from *M. malabathricum*. Figure 16S: Mass spectrum of quercetin 3-O-(6”-O-galloyl)-*β-*glucopyranoside from *M. malabathricum*. Figure 17S: Mass spectrum of kaempferol from *M. malabathricum*. Figure 18S: Mass spectrum of isoquercetin from *M. malabathricum*. Figure 19S: Mass spectrum of dorsteniol from *F. religiosa*. Figure 20S: Fragmentation pattern of catechin/epicatechin. Figure 21S: Fragmentation pattern of gallocatechin/epigallocatechin. Figure 22S: Fragmentation pattern of emodin. Figure 23S: Fragmentation pattern of quercetin derivatives. Figure 24S: Fragmentation pattern of kaempferol.
