# Supplementary material for: Antidiabetic, Antimicrobial, and Molecular Profiling of Selected Medicinal Plants
Source: Evid Based Complement Alternat Med. 2021 May 6;2021:5510099. doi: 10.1155/2021/5510099 (PMC8121587; doi:10.1155/2021/5510099)
Supplement: Supplementary Materials — Table 1S: Total phenolic and flavonoids content of different plants. Table 2S: Antioxidant ability of plant extracts. Figure 1S: Antimicrobial activity of A. catechu extracts against S. aureus ATCC 43300 and E. coli ATCC 2591. Figure 2S: MIC of ethyl acetate of A. catechu and F. religiosa against S. sonnei ATCC 25931. Figure 3S: MBC of ethyl acetate fraction of A. catechu and M. malabathricum against S. sonnei ATCC 25931. Figure 4S: Total ion chromatogram (TIC) of (a) dark blue line: A. catechu ethyl acetate fraction, (b) red line: S. robusta ethyl acetate fraction, (c) green line: A. catechu water fraction, (d) pink line: M. malabathricum ethyl acetate fraction, and (e) sky blue line: F. religiosa hexane fraction. Figure 5S: Mass spectrum of catechin or epicatechin from A. catechu. Figure 6S: Mass spectrum of gallocatechin or epigallocatechin from A. catechu. Figure 7: mass spectrum of procyanidin from A. catechu. Figure 8S: Mass spectrum of emodin from A. catechu. Figure 9S: mass spectrum of quercetin from S. robusta. Figure 10S: Mass spectrum of gossypin from S. robusta. Figure 11S: Mass spectrum of bergenin acid from S. robusta. Figure 12S: Mass spectrum of quercetin 3-O-β-D-apiofuranosyl (1 ⟶ 2)-[6-O-(3-hydroxy-3-methylglutaroyl)]-β-D-glucopyranoside from S. robusta. Figure 13S: Mass spectrum of quercetin 7-methyl ether 3-[3-hydroxy-3-methylglutaryl-(1->6)]-[apiosyl-(1->2)-galactoside] from S. robusta. Figure 14S:Mass spectrum of avicularin (quercetin 3-α -L-arabinofuranoside) from S. robusta. Figure 15S: Mass spectrum of kaempferol 3-O-α-L-arabinopyranoside from M. malabathricum. Figure 16S: Mass spectrum of quercetin 3-O-(6”-O-galloyl)-β-glucopyranoside from M. malabathricum. Figure 17S: Mass spectrum of kaempferol from M. malabathricum. Figure 18S: Mass spectrum of isoquercetin from M. malabathricum. Figure 19S: Mass spectrum of dorsteniol from F. religiosa. Figure 20S: Fragmentation pattern of catechin/epicatechin. Figure 21S: Fragmentation pattern of gallo [file 5510099.f1.docx]

**Antidiabetic, Antimicrobial and Molecular Profiling of Selected Medicinal Plants**

Babita Aryal^1^, Purushottam Niraula^1^, Karan Khadayat^1^, Bikash Adhikari^1^_,_ Dadhiram K.C^1^, Basanta Kumar Sapkota^1^, Bibek Raj Bhattarai^1^, Niraj Aryal^2^ and Niranjan Parajuli^1*^

**Table 1S** Total phenolic and flavonoids content of different plants

| **Name of the plant** | **TPC (mg GAE/gm)** | **TFC (mg QE/gm)** |  |
| --- | --- | --- | --- |
| *Acacia catechu* | 186.675 ± 2.021 | 10.24 ± 0.69 |  |
| *Shorea robusta* | 131.25 ± 5.001 | 297.17 ± 12.91 |  |
| *Melastoma malabathricum* | 76.542 ± 1.599 | 247.5 ± 12.56 |  |
| *Ficus religiosa* | 14.54 ± 0.62 | 44.67 ± 0.31 |  |

Total phenolic contain (TPC) of plant extracts are expressed in terms of gallic acid equivalent (mg GAE/gm dry weight of extract) and Total flavonoid content (TFC) of plant extracts is expressed in terms of quercetin equivalent (mg QE/gm)

**Table 2S** Antioxidant ability of plant extracts

| **Plants extract** | **IC_50_ (µg/mL)** |
| --- | --- |
| *Acacia catechu* | 84.9 ± 1.9 |
| *Ficus religiosa* | 67.4 ± 0.6 |
| *Melastoma malabathricum* | 74.9 ± 5.6 |
| *Shorea robusta* | 111.4 ± 1.1 |
| Quercetin (Standard) | 6.3 ± 1.0 |


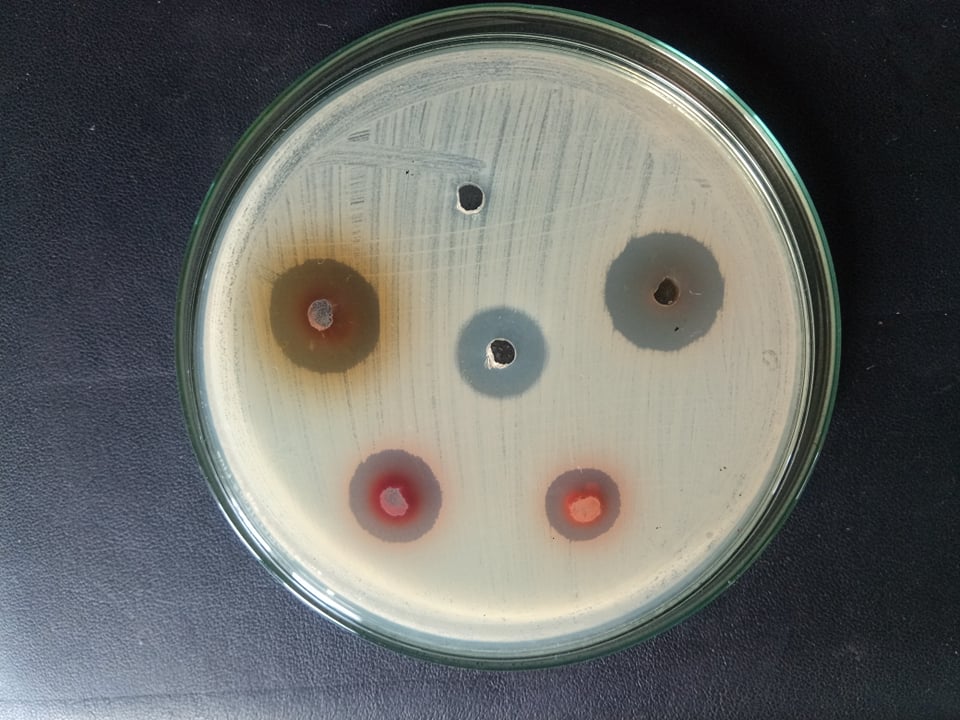

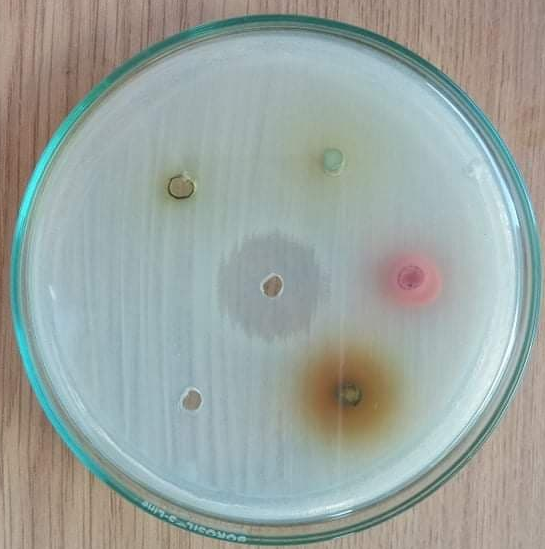


***S. aureus***

***E. coli***

**E**

**W**

**D**

**H**

**PC**

**NC**

**NC**

**D**

**E**

**H**

**W**

**PC**

(H: Hexane, D-DCM, E-Ethyl acetate, W- Water fractions of plants extract; PC: Positive control; NC: Negative control)

**Figure 1S** Antimicrobial activity of *A. catechu* extracts against *S. aureus* ATCC 43300 and *E. coli* ATCC 2591


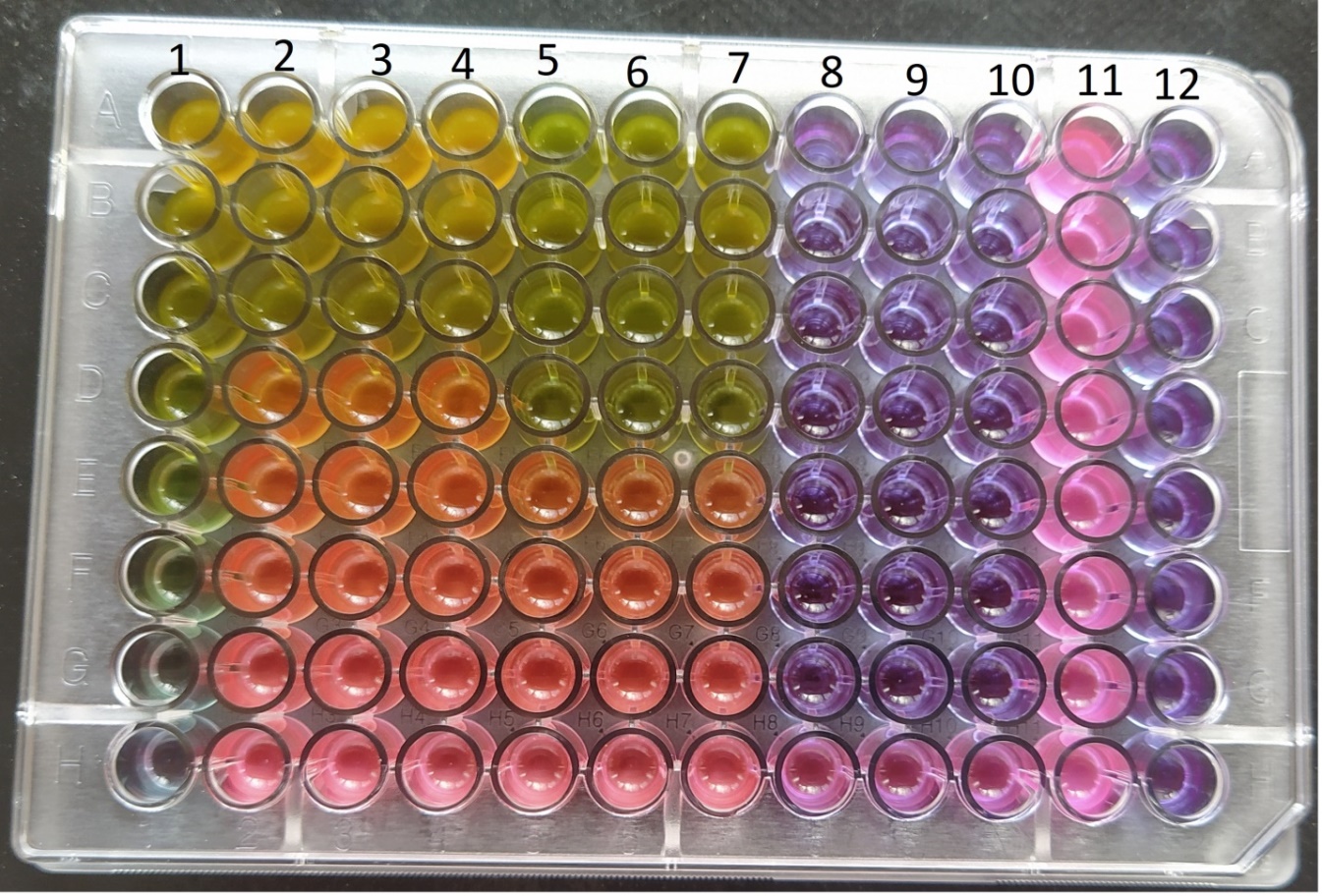


(1: *Acacia catechu* plant control; 2, 3, 4: ethyl acetate fraction of *Acacia catechu*; 5, 6, 7: ethyl acetate fraction of *Ficus religiosa*; 8, 9, 10: Positive control (Neomycin); 11: control with bacteria; 12: control without bacteria)

**Figure 2S** MIC of ethyl acetate of *A. catechu* and *F. religiosa* against *S. sonnei* ATCC 25931.


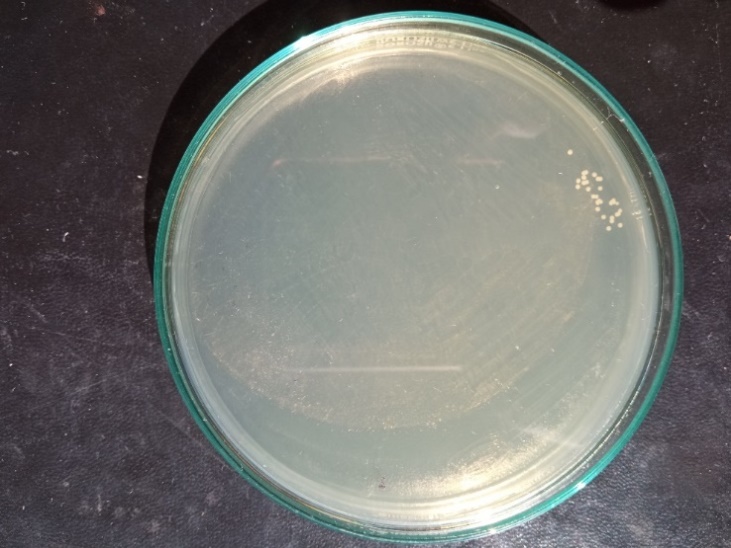

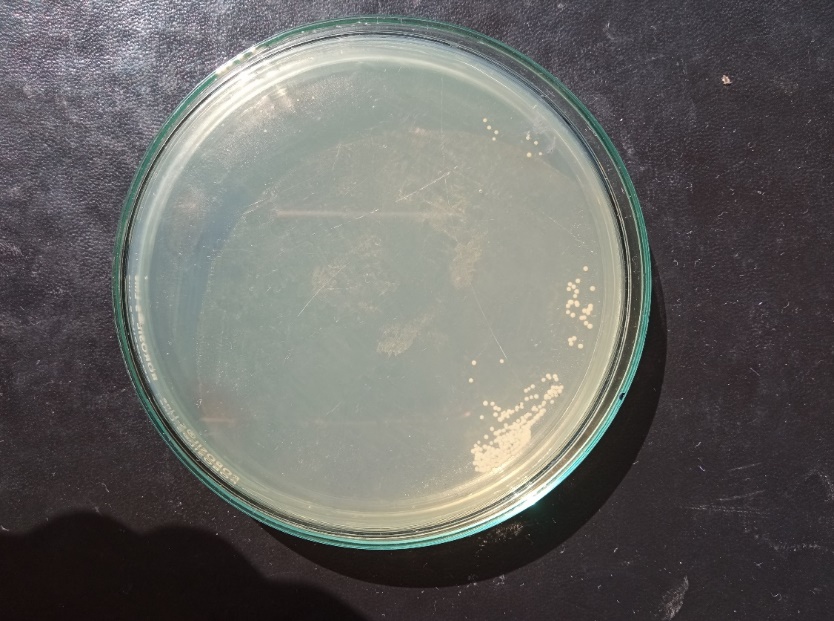


**D**

**C**

**B**

**A**

**MBC**

**MBC**

**C**

**A**

**B**

**D**

**E**

**F**

**Figure 3S** MBC of ethyl acetate fraction of *A. catechu* and *M. malabathricum* against *S. sonnei* ATCC 25931.

*
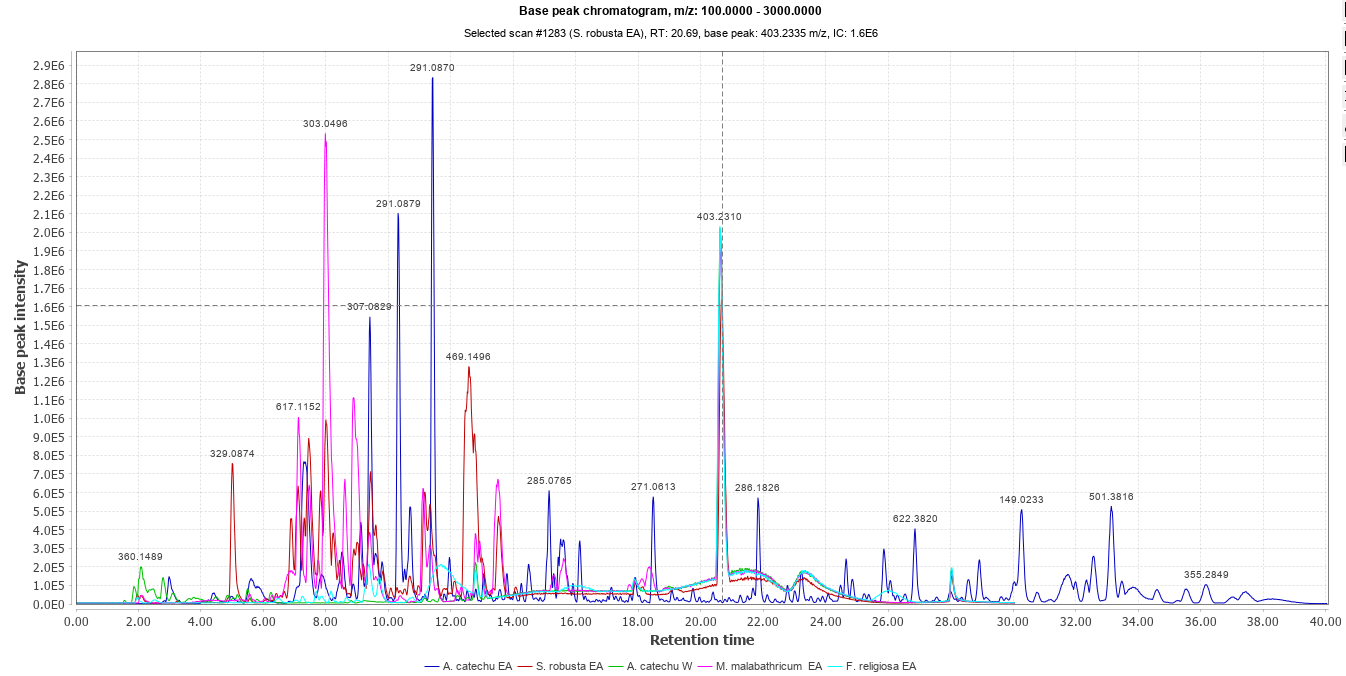
*

**Figure 4S** Total ion chromatogram (TIC) of a) Dark blue line: *A. catechu* ethyl acetate fraction, b) Red line: *S. robusta* ethyl acetate fraction c) Green line: *A. catechu* water fraction, d) Pink line: *M. malabathricum* ethyl acetate fraction and e) Sky blue line: *F. religiosa* hexane fraction.

**Figure 5S** Mass spectrum of catechin or epicatechin from *A. catechu*

**Figure 6S** Mass spectrum of gallocatechin or epigallocatechin from *A. catechu*

**Figure 7S** Mass spectrum of procyanidin from *A. catechu*


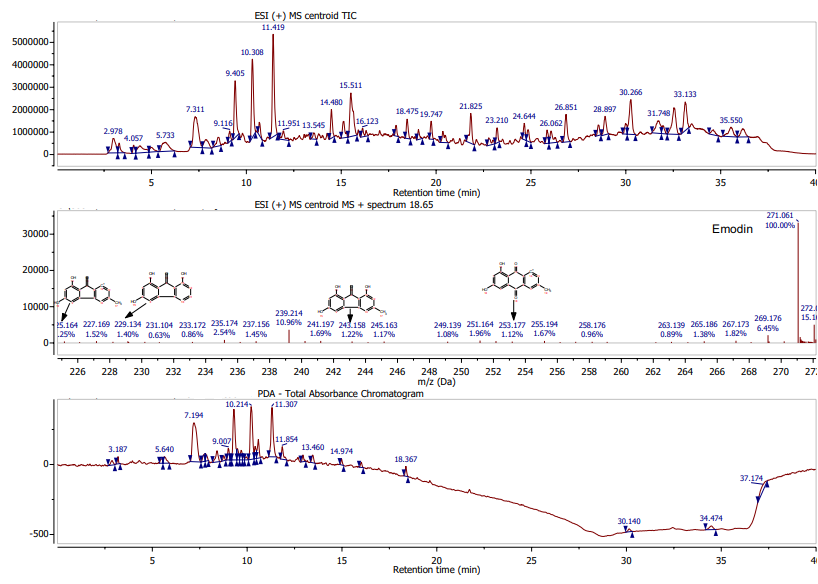


**Figure 8S** Mass spectrum of emodin from *A. catechu*


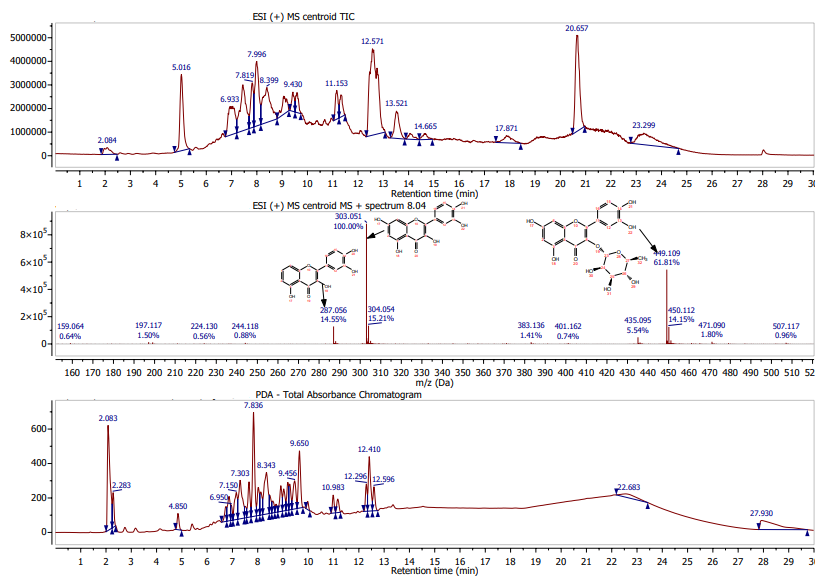
 **Figure 9S** Mass spectrum of quercetin from *S. robusta*

*
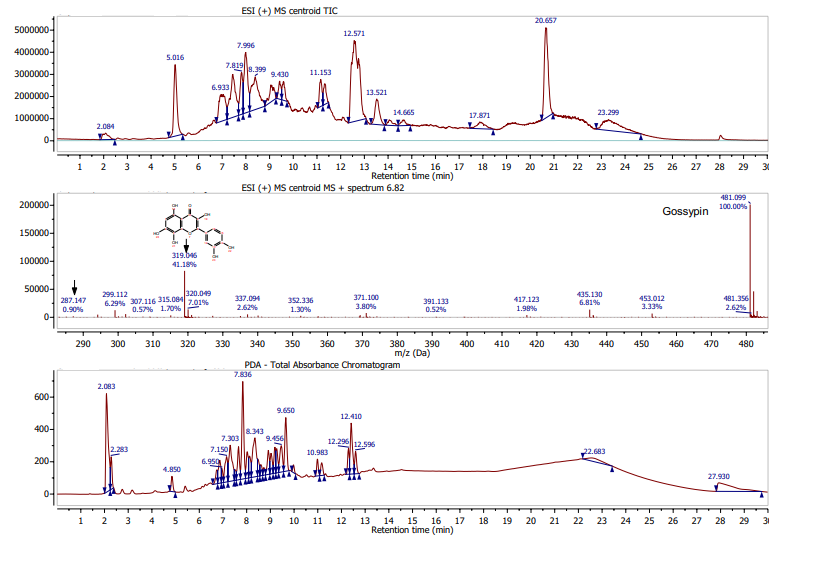
***Figure 10S** Mass spectrum of gossypin from *S. robusta*

**Figure 11S** Mass spectrum of bergenin acid from *S. robusta*

*
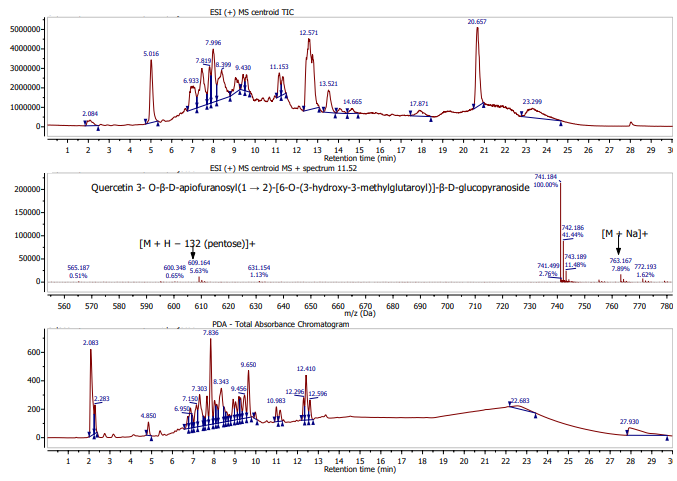
***Figure 12S** Mass spectrum of quercetin 3-O-β-D-apiofuranosyl(1 → 2)-[6-O-(3-hydroxy-3-methylglutaroyl)]-β-D-glucopyranoside from *S. robusta*


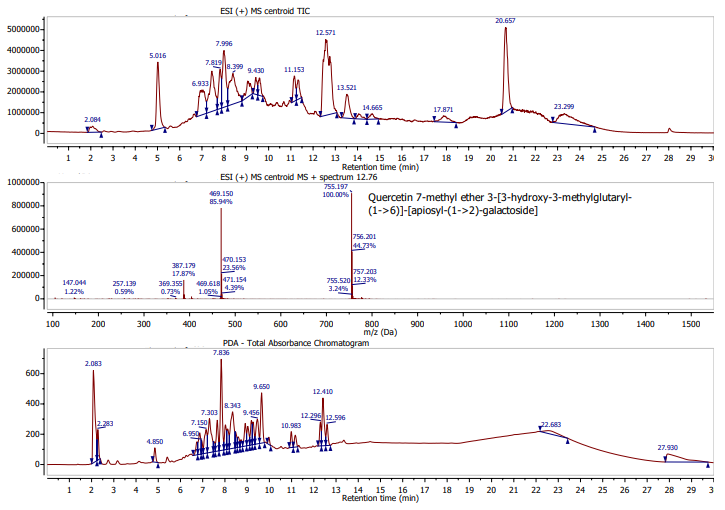


**Figure 13S** Mass spectrum of quercetin 7-methyl ether 3-[3-hydroxy-3-methylglutaryl-(1->6)]-[apiosyl-(1->2)-galactoside] from *S. robusta*


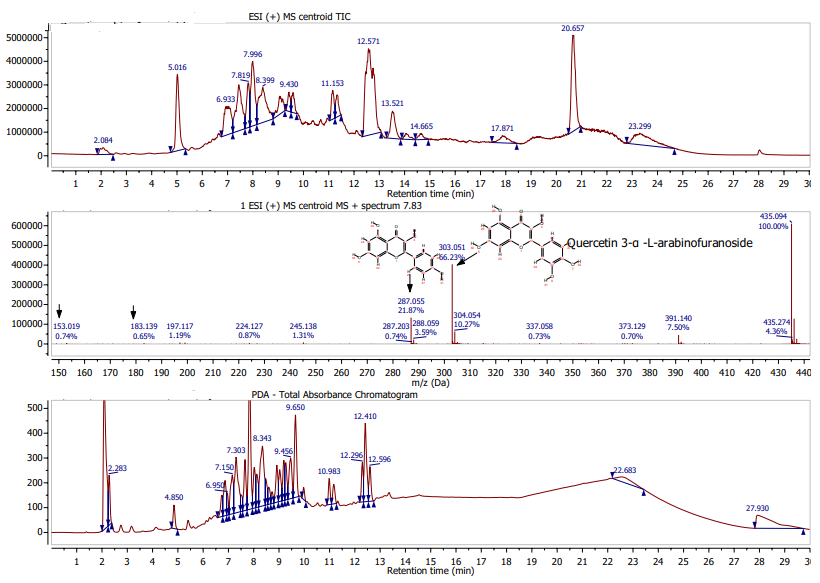


**Figure 14S** Mass spectrum of avicularin (quercetin 3-α -L-arabinofuranoside) from *S. robusta*

*
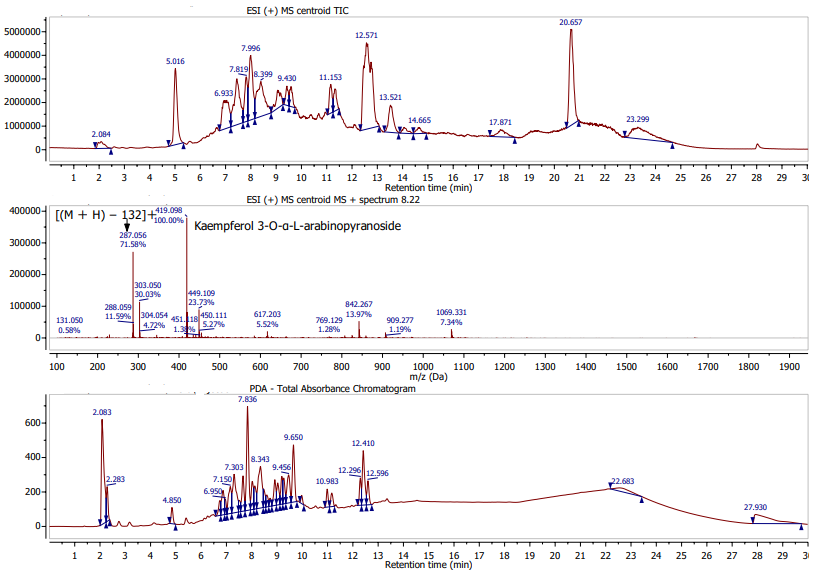
*

**Figure 15S** Mass spectrum of kaempferol 3-O-α-L-arabinopyranoside from *M. malabathricum*

*
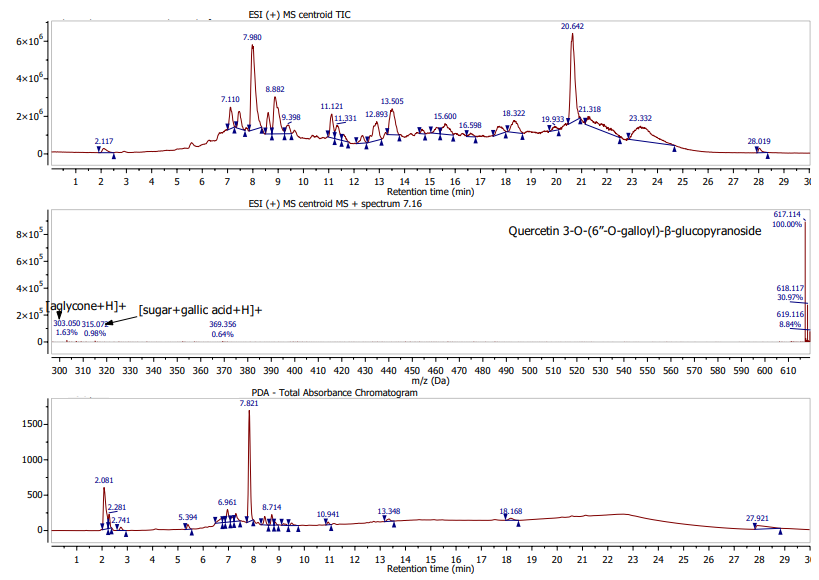
***Figure 16S** Mass spectrum of quercetin 3-O-(6’’-O-galloyl)-β-glucopyranoside from *M. malabathricum*

*
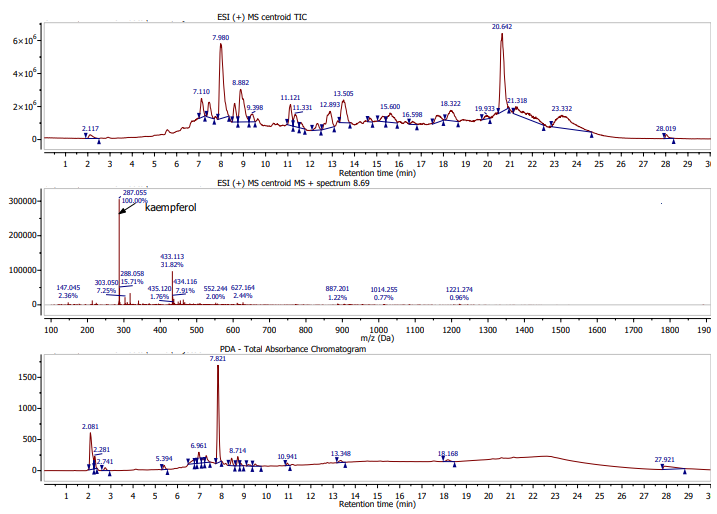
*

**Figure 17S** Mass spectrum of kaempferol from *M. malabathricum*

*
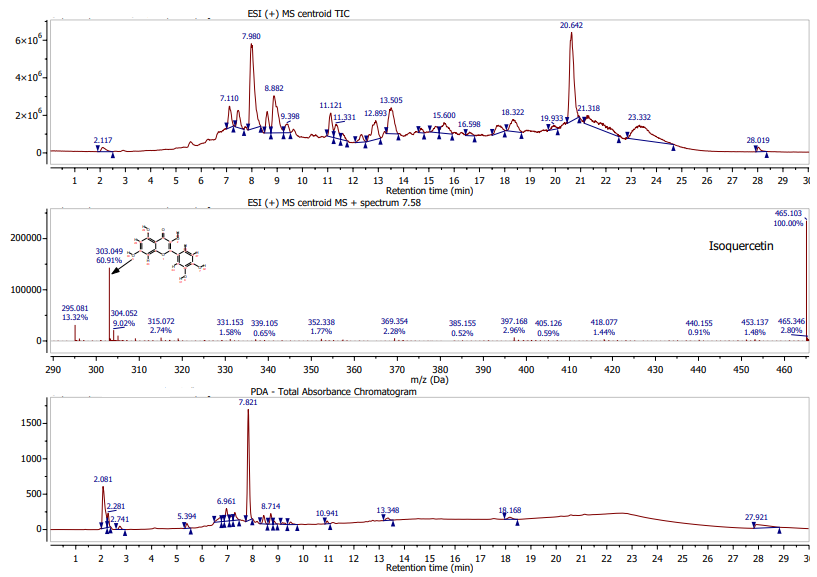
*

**Figure 18S** Mass spectrum of isoquercetin from *M. malabathricum*

*
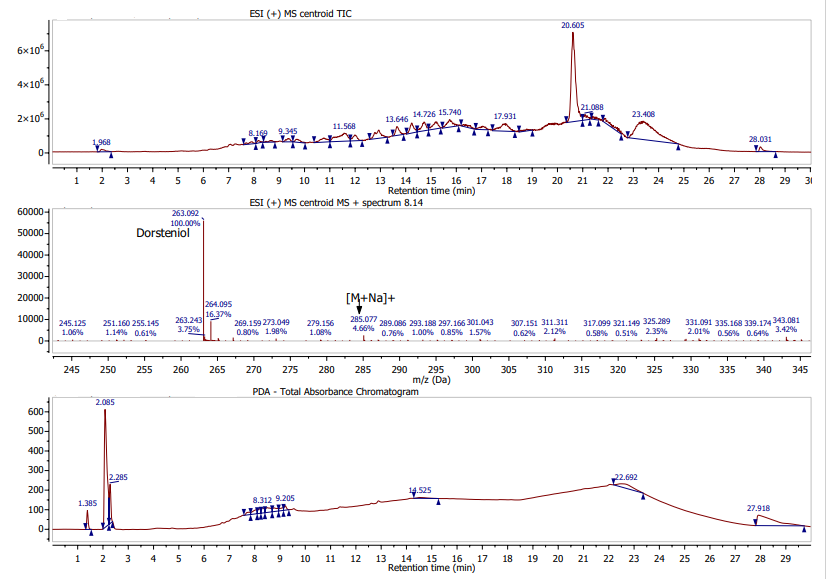
* **Figure 19S** Mass spectrum of dorsteniol from *F. religiosa*


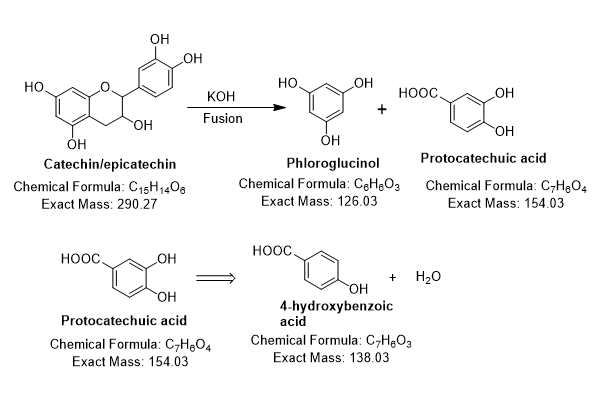
 **Figure 20S** Fragmentation pattern of catechin/epicatechin.


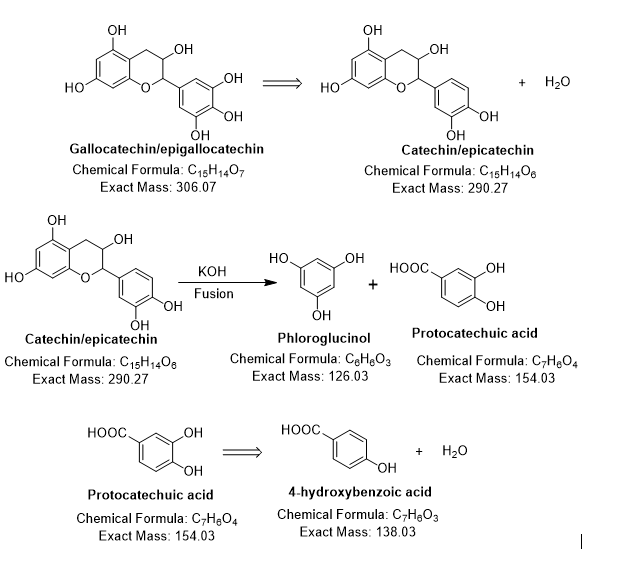


**Figure 21S** Fragmentation pattern of gallocatechin/epigallocatechin


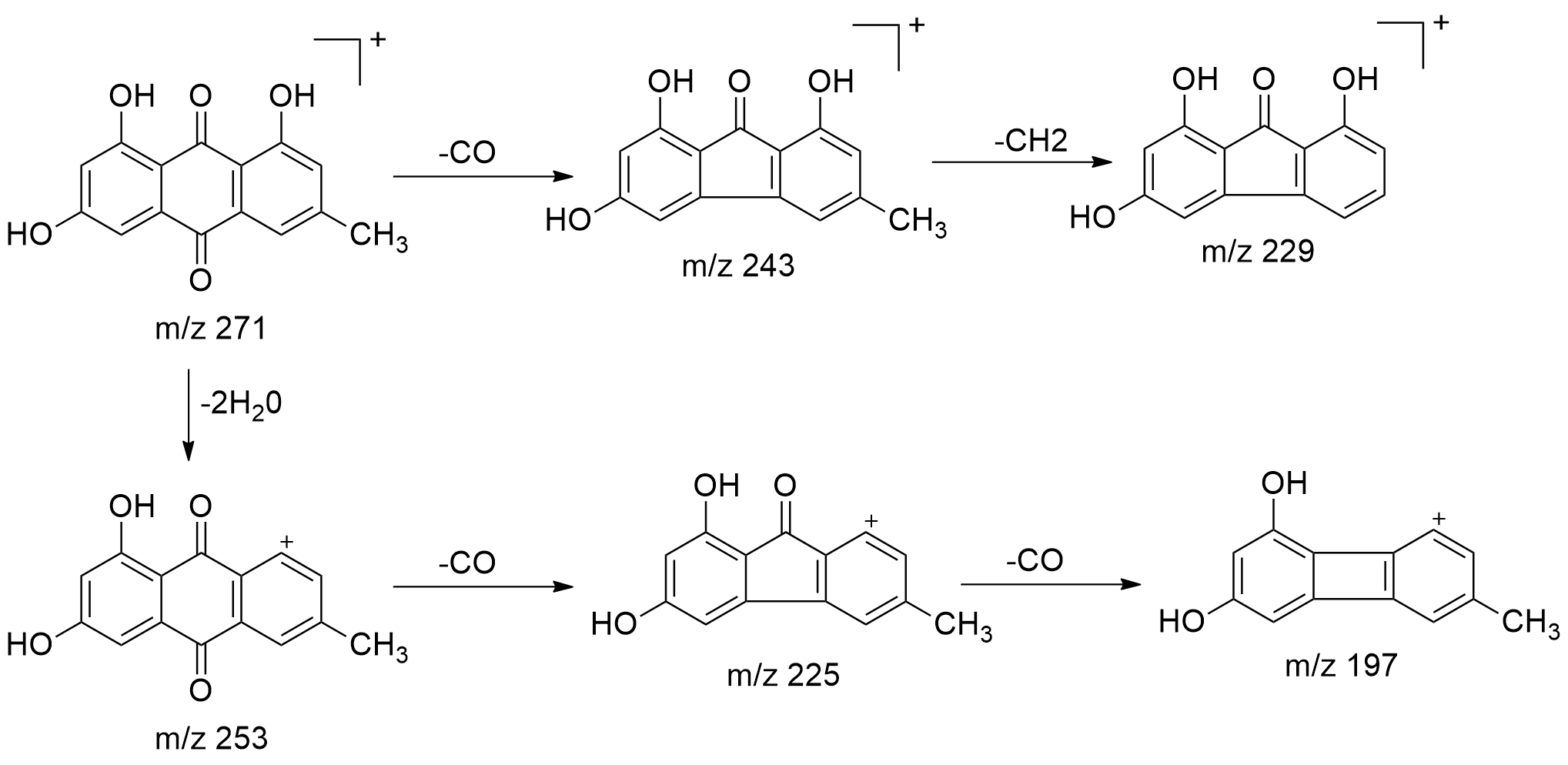
 **Figure 22S:** Fragmentation pattern of emodin.


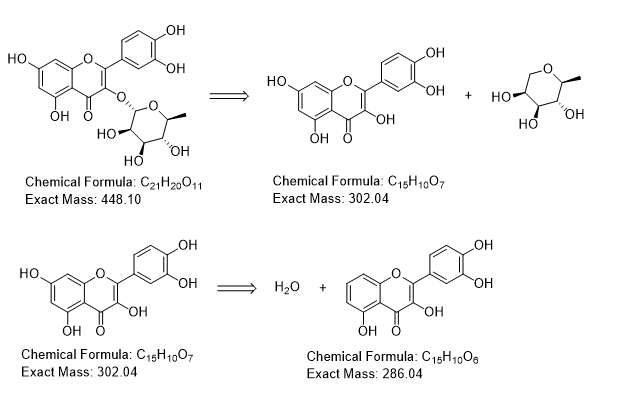
 **Figure 23S** Fragmentation pattern of quercetin derivatives.


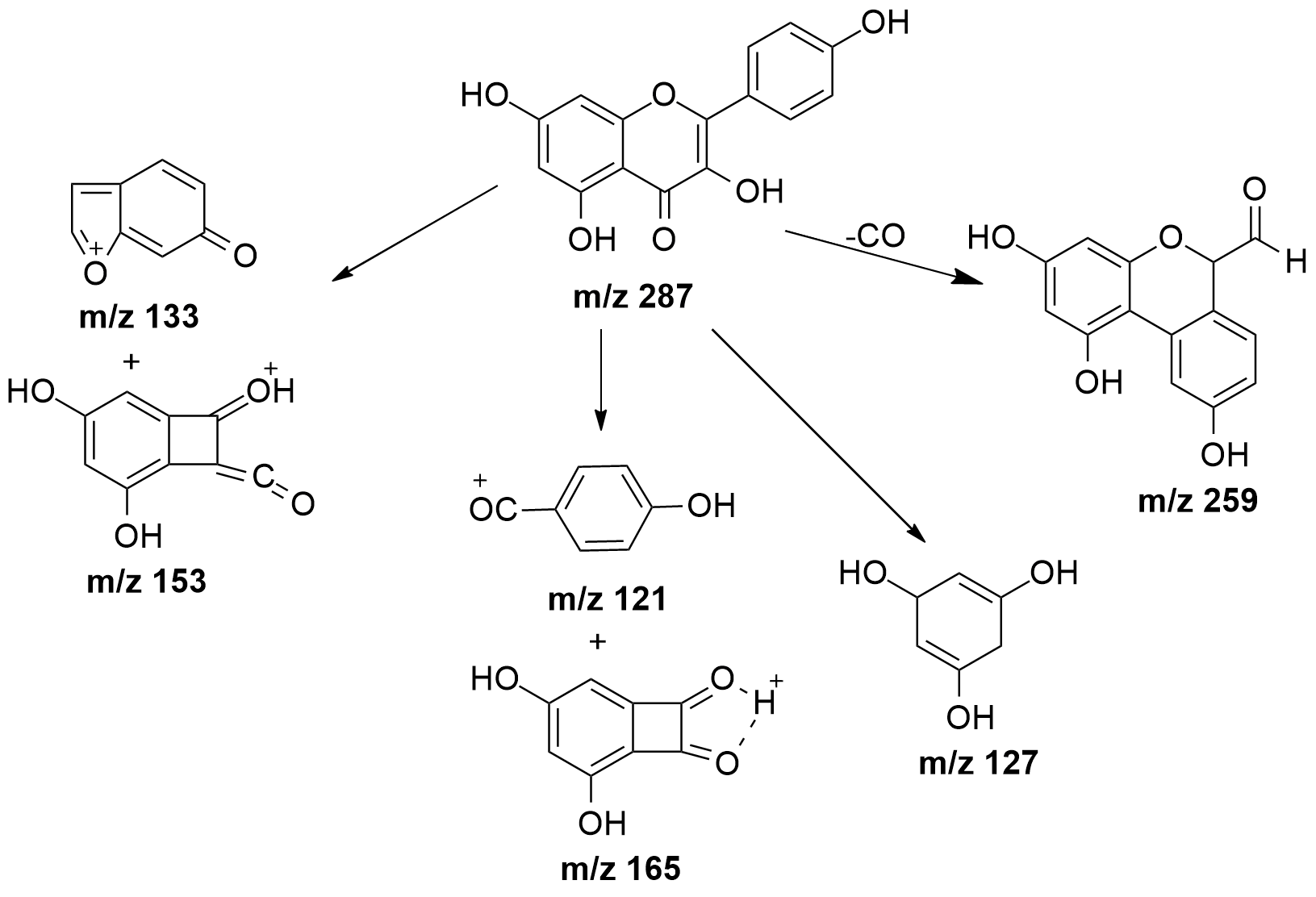
 **Figure 24S** Fragmentation pattern of kaempferol.
